# Interaction between genetic susceptibility to obesity and food intake on BMI in Finnish school-aged children

**DOI:** 10.1038/s41598-023-42430-5

**Published:** 2023-09-14

**Authors:** Heli Viljakainen, Jose V. Sorlí, Emma Dahlström, Nitin Agrawal, Olga Portolés, Dolores Corella

**Affiliations:** 1 Folkhälsan Research Center, Topeliuksenkatu 20, 00250 Helsinki, Finland; 2 Faculty of Medicine, University of Helsinki, Helsinki, Finland; 3 Department of Preventive Medicine and Public Health, University of Valencia, Valencia, Spain; 4 CIBER Fisiopatología de la Obesidad y Nutrición, Madrid, Spain; 5 Research Program for Clinical and Molecular Metabolism, Faculty of Medicine, University of Helsinki, 00290 Helsinki, Finland; 6 Department of Nephrology, University of Helsinki and Helsinki University Hospital, 00290 Helsinki, Finland

**Keywords:** Nutrition, Obesity, Genetic predisposition to disease, Nutrigenomics

## Abstract

Diet modulates the genetic risk of obesity, but the modulation has been rarely studied using genetic risk scores (GRSs) in children. Our objectives were to identify single nucleotide polymorphisms (SNPs) that drive the interaction of specific foods with obesity and combine these into GRSs. Genetic and food frequency data from Finnish Health in Teens study was utilized. In total, 1142 11-year-old subjects were genotyped on the Metabochip array. BMI-GRS with 30 well-known SNPs was computed and the interaction of individual SNPs with food items and their summary dietary scores were examined in relation to age- and sex-specific BMI z-score (BMIz). The whole BMI-GRS interacted with several foods on BMIz. We identified 7–11 SNPs responsible for each interaction and these were combined into food-specific GRS. The most predominant interaction was witnessed for pizza (*p* < 0.001): the effect on BMIz was b − 0.130 (95% CI − 0.23; − 0.031) in those with low-risk, and 0.153 (95% CI 0.072; 0.234) in high-risk. Corresponding, but weaker interactions were verified for sweets and chocolate, sugary juice drink, and hamburger and hotdog. In total 5 SNPs close to genes *NEGR1, SEC16B, TMEM18, GNPDA2,* and *FTO* were shared between these interactions. Our results suggested that children genetically prone to obesity showed a stronger association of unhealthy foods with BMIz than those with lower genetic susceptibility. Shared SNPs of the interactions suggest common differences in metabolic gene-diet interactions, which warrants further investigation.

## Introduction

Obesity is a complex condition resulting from the influence of several common genetic factors in conjunction with various environmental and social factors^1^. Several candidate gene investigations^1^ as well as many recent genome-wide association studies (GWAS) have identified hundreds of single nucleotide polymorphisms (SNPs) associated with the susceptibility to obesity^2–5^. However, the genetic variants identified by GWAS with *p* < 10^–8^ explain less than 10% of the variance in body mass index (BMI)^2^. Among the genetic factors, SNPs within the *FTO* gene have been the most associated with obesity-related phenotypes in GWAS conducted in various populations^3–7^.

The role of diet in cardiometabolic diseases is widely recognized^8–10^. More specifically, unbalanced diets composed of processed, energy-dense foods, can promote weight gain in all ages^11, 12^. A genetic susceptibility to obesity appears stronger in an obesogenic environment, mainly due to an energy-dense diet, than in sparse ones^13–15^, pointing to an interaction between a person’s genotype and diet. Several studies have analyzed the interaction between SNPs in the *FTO* gene and dietary factors in determining obesity-related phenotypes^16–19^. However, the genetics of obesity is also complex^20^. Apart from the rare cases of monogenic obesity, common obesity is polygenic^21, 22^. In epidemiological studies, the combined polygenic risk of obesity has been computed using several approaches. Thus, so-called genetic risk scores (GRS) summarizing the additive effect of multiple, common SNPs have been proposed^23, 24^. An obesity-related GRS summarizes the estimated effect of common genetic variants on obesity phenotype^25^. Several GRSs have been constructed and validated for obesity phenotypes^25–29^. Although these prior studies did not analyze the interaction between the GRS for obesity and diet, subsequent studies that investigated such interactions, have mainly focused on macronutrients^30, 31^ or the overall quality of the diet^32–35^ and food groups^36, 37^.

Regarding food, the interaction between GRS for obesity and individual foods has been demonstrated previously for sugar-sweetened beverages^38, 39^ and fried foods^40^: these have been associated strongly with weight gain in those with a higher genetic predisposition to obesity. However, in most cases, GRSs were used as a total score, while specific SNPs that drive the interaction with foods have been poorly investigated.

Our working hypothesis is that despite diet modulating the genetic risk of obesity (assessed by a whole GRS), only specific SNPs in the GRS, as well as specific foods, are the main drivers of such modulation. Therefore, the identification of such specific interactions in specific populations will be of special relevance to provide a more focused recommendation to promote precision health^41^.

Compared to adults, research on the interaction between food consumption and genetic predisposition to obesity in children is very scarce, although understanding it could aid early risk detection and target preventive actions efficiently^42^. Therefore, we aimed at analyzing gene-diet interactions, considering not only the whole GRS but also identifying individual SNPs that drive the interaction of specific foods and combining these into GRSs. Finally, we illustrated the interactions and the shared SNP effects to gain a deeper insight into how individuals’ susceptibility to obesity modifies the effect of food consumption on BMI.

## Results

### Participants

Background characteristics of the 1142 participants are described in Table 1 by groups with low and high genetic risk for obesity. The grouping was based on the median number of risk alleles (n = 27). The number of risk alleles varied between 17 and 27 in the low-risk group and between 28 and 39 in the high-risk group. There was a distinctive difference in BMIz and waist-to-height-ratio (WtHr) between the groups: higher BMIz and waist circumference, but not height, was observed in the high -risk group in comparison to the low-risk group. However, many demographic and lifestyle factors did not differ between the groups. Correspondingly, food consumption illustrated by three summary scores and 15 individual food items were similar between the groups. An exception was observed with the consumption of pizza, which was somewhat higher (0.63 vs. 0.53, *p* = 0.061) and had a double variation in the high compared with the low-risk group.

**Table 1:** Background characteristics of the participants in low- and high-risk groups, using the median number (= 27) of risk alleles as the cut-off, reported as mean (SD), if not indicated otherwise.

n	Low risk		High risk		*p* value
	603		539		
	Mean	SD	Mean	SD	
Number of risk alleles					< 0.001
Sex, n (%)	24.8	(2.1)	30.3	(2.1)	0.947^a^
Girl	302	(50.1%)	271	(50.3%)	
Boy	301	(49.9%)	268	(49.7%)	
Puberty phase, n (%)^b^					0.591
Prepubertal	249	(45.4%)	232	(47.6%)	0.500^a^
Pubertal	296	(54.0%)	250	(51.3%)	
Postpubertal	3	(0.5%)	5	(1.0%)	
Age, y	11.30	(0.2)	11.30	(0.2)	0.712
BMI z-score	0.21	(1.0)	0.47	(1.0)	< 0.001
Waist-to-height ratio	0.44	(0.04)	0.45	(0.05)	0.001
Maternal SES, n (%)^c^					0.161^a^
Upper-level	175	(29.0%)	154	(28.8%)	
Lower-level	261	(43.3%)	213	(39.5%)	
Manual workers	59	(9.8%)	62	(11.5%)	
Self-employed	7	(1.2%)	7	(1.2%)	
Students	43	(7.1%)	52	(9.6%)	
Housewife	16	(2.7%)	21	(3.9%)	
Other	0	(0.0%)	3	(0.3%)	
Leisure-time physical activity, h/week^d^	6.8	(2.7)	6.6	(2.6)	0.315
Mean sleep duration for week, h/night^e^	9.8	(0.7)	9.8	(0.6)	0.940
Eating habit group, n (%)^f^					0.591^a^
Healthy	69	(12.8%)	70	(14.2%)	
Fruit and vegetable avoider	233	(43.1%)	219	(44.5%)	
Unhealthy	239	(44.2%)	203	(41.3%)	
Plant consumption index, times/week^g^	12.0	(7.9)	11.9	(8.0)	0.308
Sweet treat consumption index, times/week^h^	9.9	(7.4)	9.9	(8.1)	0.882
Individual food items, times/week					
Dark bread^i^	4.7	(4.0)	4.9	(4.1)	0.484
Sweet pastry^j^	1.2	(1.4)	1.1	(1.5)	0.691
Biscuits and cookies^k^	1.8	(2.2)	1.8	(2.4)	0.921
Ice cream^l^	2.0	(2.1)	2.0	(2.2)	0.852
Sugary juice drink^m^	2.2	(2.8)	2.3	(3.3)	0.549
Sugary soft drink^n^	1.4	(2.0)	1.4	(2.0)	0.631
Sweets and chocolate^o^	1.4	(1.4)	1.4	(1.4)	0.946
Pizza^p^	0.5	(0.7)	0.6	(1.2)	0.061
Hamburger and hotdog^q^	0.6	(0.8)	0.6	(1.3)	0.148
Milk and sourmilk^r^	10.5	(5.0)	10.5	(5.0)	0.985
Cooked vegetables^s^	1.6	(2.4)	1.8	(2.6)	0.471
Fresh and grated vegetables^t^	5.7	(4.3)	5.6	(4.2)	0.701
Fruit and berries^u^	4.6	(3.8)	4.5	(3.7)	0.660
Juice^v^	3.5	(3.8)	3.5	(3.7)	0.980
Salty snacks^w^	1.0	(1.4)	1.0	(1.1)	0.764
Water^x^	9.7	(4.9)	9.9	(5.0)	0.495

^a^Chi-Square, Missing values in low- and high-group, respectively: ^b^n = 55 and n = 52, ^c^n = 42 and n = 27, ^d^n = 4 and n = 5, ^e^n = 39 and n = 33, ^f^n = 62 and n = 47, ^g^n = 13 and n = 16, ^h^n = 13 and n = 16, ^i^n = 9 and n = 8, ^j^n = 10 and n = 2, ^k^n = 8 and n = 2, ^l^n = 7 and n = 4, ^m^n = 36 and n = 18, ^n^n = 11 and n = 8, ^o^n = 17 and n = 13, ^p^n = 11 and n = 6, ^q^n = 9 and n = 5, ^r^n = 9 and n = 3, ^s^n = 10 and n = 7, ^t^n = 7 and n = 6, ^u^n = 10 and n = 7, ^v^n = 7 and n = 4, ^w^n = 9 and n = 5, ^x^n = 9 and n = 1.

### Foods with an interaction with the whole GRS

Interactions of dietary summary scores/individual food items with whole BMI-GRSs on BMIz are shown in Table 2. We witnessed interactions for five individual food items: dark bread, biscuits and cookies, sugary juice drink, sweets and chocolate, pizza, and milk and sour milk with at least one BMI-GRS *p* < 0.15. When using dichotomous BMI-GRS e.g., (low vs. high-risk group), an additional interaction was identified for hamburger and hotdog. Details of the interactions are shown in Supplementary Table 1. There was no interaction between any of the dietary summary scores and BMI-GRS on BMIz.

**Table 2:** *P* value for interaction between BMI-GRSs and food items regarding BMIz. The analyses were adjusted for sex, leisure-time physical activity, sleep duration and 1st and 2nd principal coordinates (PC) for population structure.

	Unweighted BMI-GRS^a^	BMI-GRS_Speliotes_^b^	BMI-GRS_Fin-HIT_^c^	BMI-GRS_ratio_^d^
Summary indexes				
Eating habit group	0.783	0.703	0.919	0.207
Plant consumption index, times/week	0.840	0.648	0.993	0.604
Sweet treat consumption index, times/week	0.603	0.537	0.753	0.347
Individual food items, times/week				
Dark bread	**0**.**099**	0.200	0.240	0.813
Sweet pastry	0.972	0.772	0.541	0.273
Biscuits and cookies	0.411	0.277	**0**.**149**/**0**.**112**^e^	0.449
Ice cream	0.311	0.389	0.233	0.263
Sugary juice drink	0.190	**0**.**131**	0.621	0.451
Sugary soft drink	0.669	0.691	0.558	0.342
Sweets and chocolate	**0**.**080**	**0**.**060**	0.575	0.386
Pizza	**0**.**017**	**0**.**078**	**0**.**058**	0.309
Hamburger and hotdog	0.756	0.878	0.203	**0**.**153**/**0**.**079**^**e**^
Milk and sourmilk	0.567	0.210	**0**.**017**	**0**.**040**
Cooked vegetables	0.276	0.612	0.152/0.186^e^	0.165
Fresh and grated vegetables	0.637	0.770	0.733	0.637
Fruit and berries	0.447	0.367	0.612	0.501
Juice	0.434	0.393	0.987	0.171
Salty snacks	0.669	0.659	0.963	0.562
Water	0.771	0.744	0.978	0.637

^a^Sum of risk alleles.

^b^Effect sizes from Speliotes et al.^21^.

^c^Effect sizes from Fin-HIT^52^.

^d^Effect size is the ratio between Fin-HIT and Speliotes.

^e^Dicotomized BMI-GRS.

Significant values are in [bold].

### Specific SNP × food modulation: SNPs driving the interaction

The identified seven food items were further explored for interactions at individual SNP levels. SNPs with the same direction and *p* < 0.2 were included in the food-specific GRS (Supplementary Table 2). In total, dark bread had 10, biscuits and cookies 7, sugary juice drink 7, sweets and chocolate 10, pizza 11, hamburger and hotdog 7, and milk and sour milk 12 interacting SNPs.

### Food-specific GRS and their interactions

**Figure 1:**
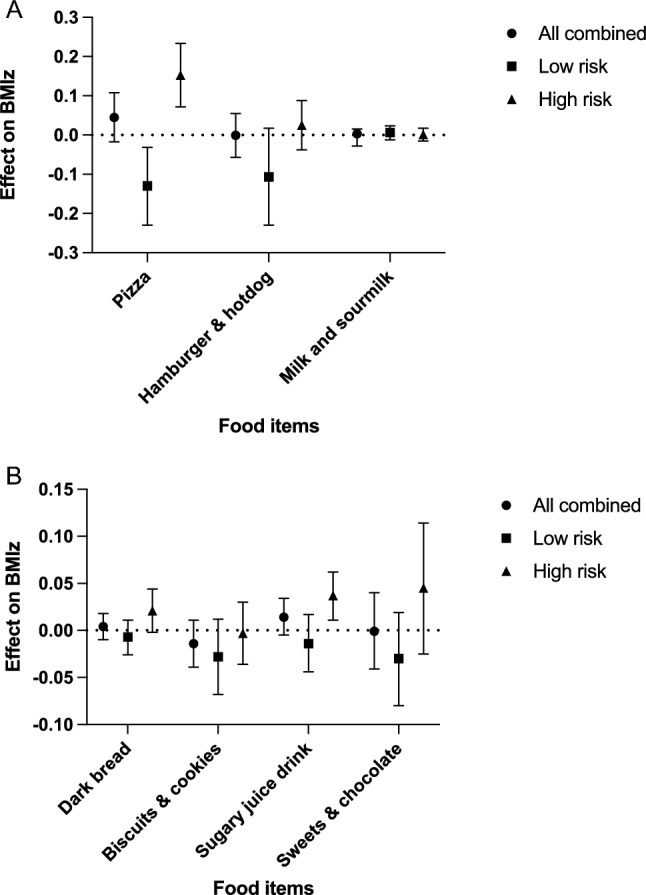
Food-specific GRS and their confirmed interactions. The results are divided into two panels for clarity and based on the food-specific GRS presented with b-coefficients with 95% CI. The most predominant interaction was marked for pizza. Other notable interactions were sugary juice drink, hamburger and hotdog, and sweets and chocolate but without formal significance. The figure was made with PRISM version 9.5.0 (https://www.graphpad.com/).

The associations of food-specific GRS, food intake, and their interaction on BMIz were tested in two models (Table 3): model 1 was adjusted only for sex, while model 2 was additionally adjusted for physical activity and sleep duration (fully adjusted). The fully adjusted interactions were further illustrated in Fig. 1. The interactions were validated for pizza, sweets and chocolate, sugary juice drink, and hamburger and hotdog when the adjusted mean effect sizes  differed between the low- and high-risk groups.

**Table 3:** The associations of food-specific GRS, food intake and their interaction with BMIz in two models.

Food	Model		b	SEM	Beta	t	*p* value	95% CI for b	
Dark bread	1	Risk allele score (rs1514175, rs543874, rs2867125, rs13107325, rs206936, rs7127684, rs7138803, rs1421085, rs571312 and rs3810291)	0.038	0.024	0.072	1.565	0.118	− 0.01	0.085
		Dark bread	− 0.088	0.032	− 0.366	− 2.785	0.005	− 0.15	− 0.026
		Interaction	0.011	0.004	0.388	2.86	0.004	0.004	0.019
	2	Risk allele score (rs1514175, rs543874, rs2867125, rs13107325, rs206936, rs7127684, rs7138803, rs1421085, rs571312 and rs3810291)	0.036	0.024	0.068	1.489	0.137	− 0.011	0.083
		Dark bread	− 0.086	0.031	− 0.357	− 2.742	0.006	− 0.147	− 0.024
		Interaction	0.011	0.004	0.398	2.957	0.003	0.004	0.019
Biscuits and cookies	1	Risk allele score (rs543874, rs11676272, rs3817334, rs7138803, rs10134820, rs2241423 and rs571312)	0.051	0.026	0.079	1.975	0.049	0	0.102
		Biscuits and cookies	− 0.094	0.05	− 0.22	− 1.877	0.061	− 0.192	0.004
		Interaction	0.018	0.01	0.208	1.741	0.082	− 0.002	0.038
	2	Risk allele score (rs543874, rs11676272, rs3817334, rs7138803, rs10134820, rs2241423 and rs571312)	0.047	0.026	0.072	1.822	0.069	− 0.004	0.097
		Biscuits and cookies	− 0.101	0.05	− 0.237	− 2.042	0.041	− 0.198	− 0.004
		Interaction	0.018	0.01	0.215	1.82	0.069	− 0.001	0.038
Sugary juice drink	1	Risk allele score (rs2815752, rs1514175, rs543874, rs2867125, rs10938397, rs7138803 and rs1421085)	0.073	0.021	0.13	3.423	0.001	0.031	0.114
		Sugary juice drink	− 0.059	0.041	− 0.181	− 1.443	0.149	− 0.14	0.021
		Interaction	0.011	0.006	0.231	1.819	0.069	− 0.001	0.022
	2	Risk allele score (rs2815752, rs1514175, rs543874, rs2867125, rs10938397, rs7138803 and rs1421085)	0.069	0.021	0.123	3.268	0.001	0.027	0.11
		Sugary juice drink	− 0.068	0.041	− 0.208	− 1.671	0.095	− 0.148	0.012
		Interaction	0.012	0.006	0.263	2.089	0.037	0.001	0.023
Sweets and chocolate	1	Risk allele score (rs2815752, rs543874, rs2867125, rs11676272, rs10938397, rs2112347, rs10134820, rs1421085, rs571312 and rs2287019)	0.04	0.023	0.078	1.726	0.085	− 0.005	0.086
		Sweets and chocolate	− 0.285	0.122	− 0.416	− 2.345	0.019	− 0.523	− 0.046
		Interaction	0.032	0.013	0.442	2.457	0.014	0.007	0.058
	2	Risk allele score (rs2815752, rs543874, rs2867125, rs11676272, rs10938397, rs2112347, rs10134820, rs1421085, rs571312 and rs2287019)	0.039	0.023	0.075	1.689	0.091	− 0.006	0.084
		Sweets and chocolate	− 0.302	0.12	− 0.441	− 2.506	0.012	− 0.538	− 0.065
		Interaction	0.033	0.013	0.452	2.539	0.011	0.008	0.059
Pizza	1	Risk allele score (rs2815752, rs543874, rs2867125, rs11676272, rs887912, rs10938397, rs2112347, rs7127684, rs7138803, rs1421085 and rs3810291)	0.031	0.017	0.069	1.814	0.07	− 0.002	0.063
		Pizza	− 0.865	0.225	− 0.816	− 3.849	< 0.001	− 1.307	− 0.424
		Interaction	0.08	0.019	0.89	4.154	< 0.001	0.042	0.117
	2	Risk allele score (rs2815752, rs543874, rs2867125, rs11676272, rs887912, rs10938397, rs2112347, rs7127684, rs7138803, rs1421085 and rs3810291)	0.03	0.017	0.067	1.776	0.076	− 0.003	0.062
		Pizza	− 0.908	0.223	− 0.856	− 4.073	< 0.001	− 1.345	− 0.47
		Interaction	0.082	0.019	0.919	4.323	< 0.001	0.045	0.12
Hamburger and hotdog	1	Risk allele score (rs2815752, rs543874, rs2867125, rs11676272, rs10938397, rs10134820 and rs1421085)	0.068	0.023	0.108	2.988	0.003	0.023	0.113
		Hamburger and hotdog	− 0.308	0.155	− 0.327	− 1.986	0.047	− 0.613	− 0.004
		Interaction	0.05	0.024	0.349	2.107	0.035	0.003	0.097
	2	Risk allele score (rs2815752, rs543874, rs2867125, rs11676272, rs10938397, rs10134820 and rs1421085)	0.07	0.023	0.111	3.085	0.002	0.025	0.114
		Hamburger and hotdog	− 0.335	0.154	− 0.356	− 2.181	0.029	− 0.636	− 0.034
		Interaction	0.052	0.024	0.363	2.215	0.027	0.006	0.099
Milk and sourmilk	1	Risk allele score (rs1514175, rs543874, rs11676272, rs10938397, rs206936, rs2030323, rs3817334, rs7138803, rs2241423, rs12444979, rs1421085 and rs571312)	0.1	0.034	0.209	2.941	0.003	0.033	0.166
		Milk and sourmilk	0.023	0.034	0.115	0.674	0.501	− 0.043	0.089
		Interaction	− 0.002	0.003	− 0.126	− 0.7	0.484	− 0.008	0.004
	2	Risk allele score (rs1514175, rs543874, rs11676272, rs10938397, rs206936, rs2030323, rs3817334, rs7138803, rs2241423, rs12444979, rs1421085 and rs571312)	0.109	0.034	0.229	3.249	0.001	0.043	0.175
		Milk and sourmilk	0.038	0.034	0.191	1.129	0.259	− 0.028	0.104
		Interaction	− 0.003	0.003	− 0.188	− 1.052	0.293	− 0.009	0.003

Model 1: adjusted for sex, 2: additionally adjusted for leisure-time physical activity and sleep duration.

The most predominant interaction was marked for pizza: it associated inversely with b − 0.130 (95% CI − 0.23; − 0.031) with BMIz in those with low GRS, while positively with b 0.153 (95% CI 0.072; 0.234) with BMIz in those having high GRS. Sugary juice drink followed the same pattern with the exception that the association among the low GRS group did not reach formal significance. Significant interactions were noted for hamburger and hotdog (*p* = 0.027) and sweets and chocolate (*p* = 0.011): the verification followed the same pattern but without formal significance.

### Shared SNPs

**Figure 2:**
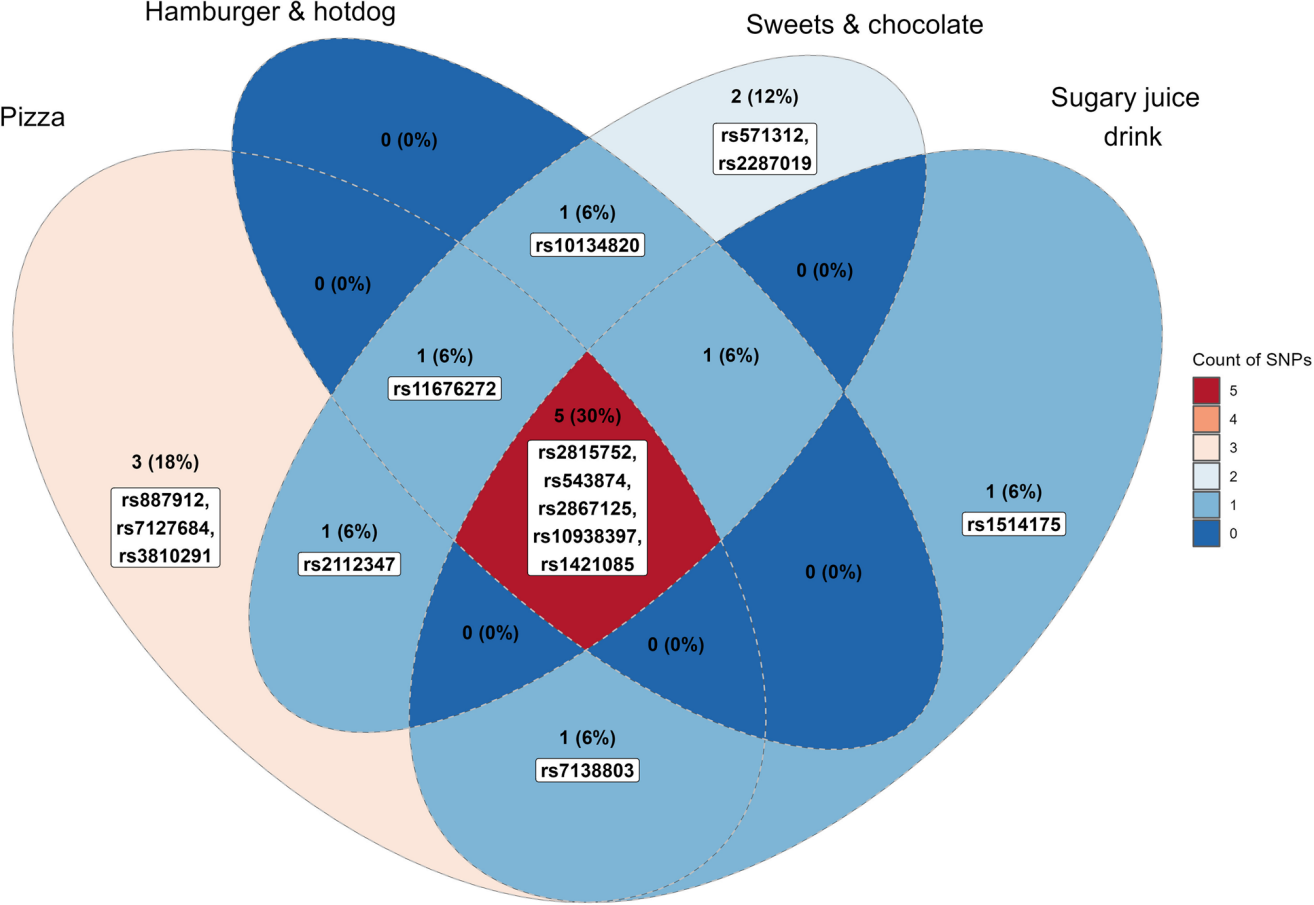
Venn diagram of shared SNPs by food items. Five (33%) SNPs interactions (in red intersection) were shared between pizza, sweets and chocolate, sugary juice drink, and hamburger and hotdog. The figure was made through R version 4.2.2 (https://posit.co/products/open-source/rstudio/).

Figure 2 illustrates the shared SNPs between the four food items. In total, we identified 15 out of 30 SNPs presenting an interaction. Interestingly, 33% of the SNPs were shared between pizza, sweets and chocolate, sugary juice drink, and hamburger and hotdog. These SNPs are close to the following genes (expressed in high magnitudes in these tissues): *NEGR1* (brain), *SEC16B* (liver/pancreas), *TMEM18* (bone), *GNPDA2* (non-specific), and *FTO* (non-specific). The description of the SNPs and genes is presented in Supplementary Table 3.

## Discussion

Initially, we observed interactions between the whole BMI-GRS and certain foods on BMIz in school-aged children from Finland. Further investigations demonstrated that each interaction was driven by 7–11 SNPs. When combining these SNPs into food-specific GRS we verified an interaction for pizza, sweets and chocolate, sugary juice drink, and hamburger and hotdog. Thus, children bearing more risk alleles for obesity showed a stronger association of weight-promoting foods on BMI than those with fewer obesity-risk alleles. Importantly, there were no differences in the consumption frequency of these foods between the groups with varying genetic susceptibility, suggesting that the effect originates from a different kind of response to food than from a difference in consumption pattern. No interactions were observed for dietary summary scores describing overall eating habits or summary food scores for sugary foods or fruits and vegetables.

The health profile of the interacting foods was considered weight-promoting based on earlier studies due to their energy-dense-nutrient-poor characteristics^43^. The most predominant interaction was observed with pizza on BMIz, e.g., a positive association with BMIz in the high-risk group while opposite in the low-risk group. Pizza consumption is the top contributor for intakes of total energy, saturated fat and sodium in US children and teens, with a daily consumption frequency of 20%^44^. In our study, once a week/in two weeks were the most common consumption patterns of pizza (> 70%). In a systematic review^45^, *TMEM18* and *FTO* were linked with total energy and fat intakes, thus partly supporting our findings.

In total, we identified 15 out of 30 SNPs as being responsible for the observed interactions for pizza, sweets and chocolate, sugary juice drink, and hamburger and hotdog. Interestingly, 33% of the interacting SNPs were shared between the foods. These included SNPs in or near genes *NEGR1* (rs2815752), *SEC16B* (rs543874), *TMEM18* (rs2867125), *GNPDA2* (rs10938397) and *FTO* (rs1421085). Except for *TMEM18*, the other SNPs were previously shown to drive the interaction of fried foods, e.g., any deep-fried foods enjoyed at home or away from home on BMI in three US cohorts^40^, while an independent effect was noted only for *FTO*. The *FTO* (rs1421085) gene has been associated repeatedly with various obesity phenotypes in different study designs and populations^46^ (Supplementary Table 3), and its expression is aggregated in primary adipocytes^47^. The variant rs1421085 in the first intron of the *FTO* gene regulates the adipocyte-thermogenesis pathway by interacting with other genes (*ARID5B, IRX3, and IRX5*)^47^. Previous reports have witnessed multiple interactions of *FTO* variant rs1421085 with the intake of fiber^48^, dietary variation, alcohol consumption, and sedentary behaviors on BMI among adults^49^.

Our unique finding concerned *TMEM18* (rs2867125), which has been associated with pediatric BMI^50, 51^, but here it presented an interaction with several foods. The contribution of any GRS or SNP may vary with age and in different stages of life^13^. The total BMI-GRS used here was significantly associated with BMIz and explained 3.7% of the variance in children^52^, which is somewhat higher than reported in adults^22^. Studies looking at genetic interaction with diet on BMI in pediatric cohorts are scarce^51^ but informative, since food consumption is more naïve and less affected by social acceptance in children than in older age groups. Thus, our results on *TMEM18* may imply that the BMI trajectory in childhood is modified by the food intake, e.g., most likely energy-rich foods provide more support for growth.

Although the weight and waist differed by the genetic susceptibility to obesity; other lifestyle factors including sleep duration, and leisure-time physical activity (LTPA) were similar between the groups. Furthermore, sleep duration and LTPA only marginally affected the results, suggesting that the interaction was independent of these factors. Certain risk variants of *FTO* (rs9939609)*, TMEM18* (rs4854344), and *NRXN3* (rs10146997) have been reported to increase the vulnerability to metabolic conditions in children under sleep deprivation^53^, thus interacting with lifestyle factors, but that was not observed here.

Sugary juice drinks are widely consumed among young Finnish children and enjoyed with a snack, instead of milk or juice at meals^54, 55^. The healthiness of sugary juice drinks is frequently discussed as the dilute berry-derived squash contains mainly sugar and provides energy, but barely nutrients. Frequent sugary juice drinkers will likely evolve with time into consumers of carbonated sugary-sweated beverages (SSB), which are deemed as weight-promoting foods^56^. Additionally, two earlier studies have demonstrated SSB to interact with the obesity-prone genotype^38, 39^. Similar to our finding on sugary juice drink, the reported magnitude of association between SSB and BMI was greater among those genetically prone to obesity, implying that the downstream effects after consuming SSB differ between the individual, making obese-prone more vulnerable to weight gain. Furthermore, Brunkwall’s study^39^ highlighted that the SSB-BMI interaction was mainly driven by one SNP – rs1555543, close to gene *PTBP2,* among middle-aged Swedish individuals. The same SNP has demonstrated an interaction with smoking on BMI in the Pakistani population^57^. However, we did not observe any interaction of rs1555543 in our sample, possibly due to the young age of the participants.

The ultimate strength of the study is that we used a cohort of school-aged children whose food consumption is likely less affected by social acceptance. Although mis- and underreporting are common challenges in dietary assessment, it is shown that amongst 11–12-year-old children that the FFQ is a valid method and independent of BMI, implying that social acceptance and desirability are less common in children than in older age groups^58^. Thus, we observed no differences in food consumption frequencies. However, we did not address portion sizes, which may differ by BMI^59^. The study was facilitated by a previously reported association of BMI-GRS and BMIz^52^, relying on 30 well-characterized SNPs. Our results may be generalized to a comparable European population with a similar socioeconomic background. Based on our earlier work^52^, using a GRS with more SNPs would most likely result in similar outcomes, as the GRSs present with corresponding associations with BMIz.

Due to the limited sample size and using the tailor-made Metabochip array only obesity SNPs were considered. Future studies with larger sample size and genome-wide coverage of SNPs are warranted for broader investigations of the interactions between genes and diet. The food frequency questionnaire (FFQ) covered 16 food items and was considered suitable for 11-year-old children to comprehend^60, 61^. However, it might have been too narrow to distinguish between foods with varying health profiles, e.g., all dairy products were considered together without considering differences in the nutrient content. Thus, we might have lost some of the information. Because power for detecting interactions is typically much lower than power for main effects, we raised the Type I error rate to 20% when assessing interactions as suggested^62, 63^. On the other hand, this might increase the chance of false positive results. However, we illustrated the association in subgroups as well.

In conclusion, the interacting foods with the genetic risk of obesity were mainly weight-promoting in Finnish children. Our results point out that children genetically prone to obesity showed a stronger association of unhealthy foods with BMIz than those with lower genetic susceptibility. Since a part of the SNPs driving the interactions were shared between the weight-promoting foods, this implies metabolic differences among genetically prone individuals, which warrants further studies in this and other geographically diverse populations.

## Methods

We have conducted a cross-sectional analysis of 1142 Finnish children. For this study, we utilized the background characteristics, genotype data, and anthropometric measurements from the Finnish Health in Teens cohort (Fin-HIT), launched in 2011 as a school-based cohort study, initially comprising 11,407 Finnish children aged between 9 and 12 years. The details of the Fin-HIT cohort are described elsewhere^64^. The Coordinating Ethics Committee of the Hospital District of Helsinki and Uusimaa has approved the study protocol (169/13/03/00/10) and written informed consent was obtained from all participants and their parents. All study procedures adhered to the 1964 Helsinki Declaration and its later amendments or comparable ethical standards.

### DNA extraction, genotyping, quality control, and generation of genetic risk score

The participants provided saliva samples by using the Oragene® DNA (OG-500) Self-Collection Kit (DNA Genotek Inc., Ottawa, Ontario, Canada). DNA was extracted using an automated protocol with the chemagic DNA Saliva Kit (PerkinElmer, Wellesley, Massachusetts). DNA samples (n = 1368) were randomly selected from the Fin-HIT cohort and subjected to genotyping with the Cardio-Metabochip (Illumina, Inc., San Diego, California) at the Finnish Institute for Molecular Medicine Technology Centre (Helsinki, Finland) as explained elsewhere^52^. The number of individuals and SNPs included in the final analysis after QC was 1142 and 125,187 with a total genotyping rate of 99.9%. BMI-based genetic risk score^25^ was based on the results of Speliotes et al. 2010^21^ but comprised 30 SNPs as rs4771122 and rs4836133 were not available and had no good proxies. Thus, within our BMI-GRS; *PTBP2* rs11165643, *TNNI3K* rs1514175, *NEGR1* rs2815752, *SEC16B* rs543874, *RBJ* rs11676272*, LRP1B rs2121279*, *TMEM18* rs2867125, *FANCL* rs887912, *CADM2* rs13078807, *ETV5* rs7647305, *GNPDA2* rs10938397, *SLC39A8* rs13107325, *FLJ35779* rs2112347, *NUDT3* rs206936, *TFAP2B* rs987237, *LRRN6C* rs10968576, *BDNF* rs2030323, *MTCH2* rs3817334, *RPL27A* rs7127684, *FAIM2* rs7138803, *PRKD1* rs10134820, *NRXN3* rs17109256, *MAP2K5* rs2241423, *GPRC5B* rs12444979, *FTO* rs1421085, *SH2B1* rs7359397, *MC4R* rs571312, *QPCTL* rs2287019, *KCTD15* rs29941, and *TMEM160* rs3810291 were considered and each increased the risk of obesity.

We summarized the number of risk alleles (unweighted) and created a weighted genetic risk score (BMI-GRS) using the score function in Plink version 1.09, which calculates an average score per non-missing SNP^52^. Besides using effect sizes of Speliotes et al. 2010^21^ (BMI-GRS_Speliotes_), also Fin-HIT effect sizes were used (BMI-GRS_Fin-HIT_), and their ratio, e.g., Fin-HIT/Speliotes (BMI-GRS_ratio_). Additionally, interactions between certain foods and individual SNPs were tested. The SNPs with the same direction of effect and *p* < 0.200 were incorporated into food-specific GRS.

### Anthropometry measurements

Children’s anthropometry, including height, waist (centimeters, cm), and weight (kilograms, kg) were measured at baseline in a standardized way by trained field workers. Children’s body mass index (BMI) (kg/m^2^) was calculated, and age- and sex-specific z-scores (BMIz) were derived based on the International Obesity Task Force (IOTF) guidelines^65^ and used as continuous variables in the analysis.

### Indicatory food items and their summary scores

Consumption frequencies of 16 food items were evaluated with a self-administered food frequency questionnaire (FFQ)^66^. For the food items, participants' ratings varied from 1; not at all, to 7; several times a day, which were recoded during analysis to scale from 0 to 14 times a week. In addition, two summary scores were created for the sweet treat index (STI) and plant consumption index (PCI) to indicate the weekly consumption frequencies of sweet treats^67^, and vegetables, fruits, and berries^68^, respectively. Our FFQ was adapted from the FFQ used in the World Health Organization’s International Health Behaviour in School-Aged Children study, which was validated and retested among school-age children in Europe^60, 61^.

Additionally, eating habits (healthy; fruit and vegetable avoider; unhealthy) were used to describe the whole diet. Those were derived with the hierarchical K-means method as explained elsewhere^66^, using the five factors obtained through factor analysis which represented 70% of the variability of the 10 selected food items.

### Other background information

Leisure-time physical activity (LTPA) and sleep habits were self-reported in the baseline questionnaire as previously described^67, 69^. LTPA duration was reported for the whole week (h/week), while sleep habits, e.g., waking and bedtime hours, separately for school days and days off. Sleep durations (with 0.5-h accuracy) were calculated, and the weighted mean for sleep duration was used in the analysis. These were used as covariates in the statistical analyses.

The questionnaire included an evaluation of pubertal development based on the Tanner stage with a pictorial assessment of breast development and pubic hair for girls and only pubic hair for boys with a scale of 1–5^70^. Due to several incomplete responses, the categorization was recoded into prepuberty (T1-2), puberty (T3-4), and postpuberty (T5) to describe the puberty phase.

Maternal occupational information at the time of the child’s birth was obtained from the Medical Birth Register maintained by the Finnish Institute for Health and Welfare and was used to describe the maternal socioeconomic status as previously described^67^. Mothers were categorized as upper-level employees, lower-level employees, manual workers, students, and others (including self-employed persons, stay-at-home mothers, unemployed persons, and pensioners). Additionally, the child’s age and sex were included.

### Statistical analyses

Background characteristics and diet were compared between groups of low and high genetic susceptibility to obesity with independent samples t-test or Chi-Square, depending on a variable. Results are presented with the mean (SD) or with n and proportion (%).

Interactions between dietary factors and BMI-GRSs/individual SNPs were tested with a linear regression model, and *p* for claiming interaction was set to < 0.15 for BMI-GRSs and < 0.2 for individual SNPs^62, 63^. The linear modeling included adjustments for covariates: sex, LTPA, mean sleep duration and 1st and 2nd principal coordinates (PC). In the case of borderline significance, the interaction was further investigated with dichotomized BMI-GRS groups stratified by the median value.

The statistical analyses were performed with IBM SPSS Statistics version 27. A significance level with 5% uncertainty was adopted.

### Supplementary Information

Supplementary Tables.

## Data Availability

Due to ethical restrictions from the coordinating ethics committee of the hospital district of Helsinki and Uusimaa (Decision Number 169/13/03/00/10) and legal GDPR restrictions, genetic data that support our findings are available upon request from the data access committee of the Fin-HIT study by contacting Dr. Heli Viljakainen (heli.viljakainen@helsinki.fi). Processed data will be available on GitHub by request: (https://github.com/Fin-HIT/Heli-Viljakainen-et-al-2023-Genetic-susceptibility-to-obesity-and-food-intake-on-BMI-in-children).
